# Supplementary material for: The discovery of novel noncoding RNAs in 50 bacterial genomes
Source: Nucleic Acids Res. 2024 Apr 22;52(9):5152–65. doi: 10.1093/nar/gkae248 (PMC11109978; doi:10.1093/nar/gkae248)
Supplement: gkae248_Supplemental_Files [file gkae248_supplemental_files.zip › Narunsky 50 Genomes RNA Motifs Files.zip!/Publication/56-D_turgidum/Figures/LRC-56-35-NC_011661_1_IGR_107_1683070_1683249.sto.pdf]

LRC-56-35-NC_011661_1_IGR_107_1683070_1683249.sto.r2r

5′-CYCACCCCC●●UAARUUARUUAGUAAGRYUCAUAAAUAAAGARA●YUU●ARA●A●UYUUU●AYCU●C●Y●AACCYYUA○●AUCACCCCUUCCY●UYY○●●R●●●AGRR●○AUUAUAUAACACUY●YUAAAA●UUGACAAURA●UAAA●YA●U●AAAAUUU
